# Nutritional status among older residents with dementia in open versus special care units in municipal nursing homes: an observational study

**DOI:** 10.1186/1471-2318-13-26

**Published:** 2013-03-14

**Authors:** Carine Aukner, Helene Dahl Eide, Per Ole Iversen

**Affiliations:** 1 Atlantis Medical College, Oslo, Norway; 2 Institute of Health, Nutrition and Management, Oslo and Akershus University College of Applied Sciences, Kjeller, Norway; 3 Department of Nutrition, Institute of Basic Medical Sciences, University of Oslo, POB 1046 Blindern, 0317 Oslo, Norway

**Keywords:** Dementia, Nursing home, Nutritional status, Open unit, Special care unit, Undernutrition

## Abstract

**Background:**

Undernutrition is widespread among institutionalised elderly, and people suffering from dementia are at particularly high risk. Many elderly with dementia live in open units or in special care units in nursing homes. It is not known whether special care units have an effect on the nutritional status of the residents. The aim of this study was therefore to examine the nutritional status of residents with dementia in both open units and in special care units.

**Methods:**

Among Oslo’s 29 municipal nursing homes, 21 participated with 358 residents with dementia or cognitive impairment, of which 46% lived in special care units. Nutritional status was assessed using the Malnutrition Universal Screening Tool and anthropometry.

**Results:**

We found no differences (p > 0.05) in risk of undernutrition, body mass index, mid-upper arm muscle circumference or triceps skinfold thickness between residents in open units and those in special care units. Residents in special care units were significantly younger and stronger when measured with a hand-grip test.

**Conclusions:**

We found no difference in nutritional status between nursing home residents with dementia/cognitive impairment in open units versus in special care units.

## Background

Undernutrition is common among residents in institutions such as hospitals and nursing homes, and its prevalence is reportedly between 10 and 70% [1-3]. There is currently no universal agreement for criteria for identification of undernutrition [4]. Therefore estimates vary in relation to methodology and study groups.

Illness is the most important cause of undernutrition in developed countries and many patients are already undernourished when they are admitted to an institution, but conditions at hospitals and care institutions may cause undernutrition to occur or progress during the stay [5]. Moreover, undernutrition has a large number of negative health consequences and it often leads to reduced health-related quality of life [5].

In Norway, approximately 70,000 people suffer from various dementia conditions and it is estimated that about 10,000 persons are affected by dementia every year. There were 41,052 places in institutions for elderly and disabled people in 2009 [6]. Of these, 39,256 places were in a nursing home which now constitutes the country’s largest institutional system. Private places make up just 10.5%.

Unwanted weight loss is common in people affected by dementia, particularly of Alzheimer’s type [7] and Grundman and colleagues found a significant association between low body mass index (BMI) and atrophy of the part of the cerebral cortex involved in control of eating behaviour [8]. Studies examining food intake of Alzheimer’s patients report, however, variable results regarding e.g. the extent of weight loss and the adequacy of their diet and/or energy intake [9,10]. The reasons for the weight loss with dementia are probably multi-factorial and the mechanism for weight loss will be different according to the type of dementia, the stage of the disease and the living situation of those affected.

Special care units were established to meet the particular needs of people affected by dementia and the goal is to offer a specially adapted environment so that residents will function normally within the limits set by the dementia condition [11]. Special care units are designed and adapted to people with dementia, and often have professional staff with special knowledge of this patient group, and there are usually more carers per resident than in a regular nursing home unit [12]. In a report from 2000 the average proportion of places in special care units in Norway was 120/1,000 residents, while the corresponding number in Oslo was 150/1,000 [13]. Based on data from 2010/2011 Kirkevold and coworkers concluded that for the special care units in Norway the number of beds and the number of staff allocated to people with dementia had been rather stable the previous 14 years [14]. They also reported that the proportion of special care units in Norwegian nursing home increased from 13.3% in 1996/1997 to 23.8% in 2010/2011. There are special criteria for the allocation of places in a special care unit in Oslo. Applicants for these places will have had (i) diagnosed a serious degree of dementia; (ii) behavioural deviations such as wandering and motor restlessness; and (iii) special requirements for security, stability and predictability in the environment that contributes to a person being sheltered from their surroundings [12]. The number of residents living per unit should be less than 12 for residents with dementia and between 4 and 8 for residents with dementia and behavioural problems, e.g. agitation [12]. The criteria for how special care units in Norway are designed and administrated are, however, vaguely defined. The lack of standardised criteria has therefore made it difficult to evaluate the effect of special care units. Studies are often not randomized and have no control groups [15]. In particular, we have little knowledge about the utility value of special care units with regard to the dementia patient’s nutritional status. The purpose of this study was therefore to examine the nutritional status of elderly residents with dementia in both open, somatic units and in special care units in municipal nursing homes in Oslo.

## Methods

We initially wanted to include all 29 municipal nursing homes in Oslo. However, three nursing homes were excluded because they had no residents meeting our inclusion criteria. From each of the remaining 26 nursing homes the aim was to randomize maximum 10 residents from open, somatic units and maximum 10 residents from special care units. To this end a numbered list of all eligible residents from every unit was provided to us from every nursing home. We then used an electronically generated list (http://www.randomization.com) of 10 random numbers to identify the study participants. Secure departments, rehabilitation units and psychiatric units were excluded. Residents who could be included had to have diagnosed dementia or a cognitive impairment as assessed by carers/nurses and/or doctors, and must have lived in the same unit for at least 6 months. All the included participants had their diagnoses of dementia/cognitive impairment given in the nursing homes’ records. These diagnoses were made either prior to admission to the nursing home or after admission. Residents were excluded from the study if they had a short life expectancy (< one month) or had aggressive and volatile behaviour rendering it impossible to perform the measurements. We did not include residents receiving enteral/parenteral nutrition. Data was collected between September 1 and November 30, 2010.

The study was approved by the Regional Committee for Medical and Health Research Ethics and it was in compliance with the Helsinki Declaration. Two different consent declarations were prepared: One for residents who could give consent themselves, and one for residents with requirements for proxy consent. For residents who could not give consent this was obtained from a next of kin or from a staff member who knew the resident well. The individual resident’s competence to give consent was evaluated in cooperation with the carer/nurse. Proxy consent was used even if the resident was competent to give consent in cases where the resident had writing difficulties or visual impairment. The nursing home was given feedback if the nutrition screening revealed that the residents were at high or medium risk of undernutrition.

Nutritional status was evaluated with the screening form Malnutrition Universal Screening Tool (MUST), BMI, percentage weight loss during the last 3–6 months, mid upper arm muscle circumference (MUAMC), triceps skinfold thickness (TSF) and hand-grip strength. MUST is a validated and recommended screening tool for detecting nutritional risk among adults in the primary health care services, and is shown to have both high reliability and practicability [4]. Based on scorings of BMI (>20 kg/m^2^ gives score 0, 18.5–20 kg/m^2^ gives score 1, < 18.5 kg/m^2^ gives score 2), percentage weight loss during the last 3–6 months (< 5% gives score 0, 5–10% gives score 1, > 10% gives score 2) and acute disease (no gives score 0, yes gives score 2), the individuals are given an overall score which categorize them to be in either low risk (overall score 0), medium risk (overall score 1) or high risk (overall score 2 or more) of malnutrition. Height was either measured standing to the nearest 0.1 cm (Seca 217 portable stadiometer), or the alternative measurements of knee height and ulna length were used. Weight was measured standing to the nearest 0.1 kg (Seca 877 class III digital floor scale). For participants who could not stand, the nursing home’s latest recorded weight was used, which was usually up to one month old. Weight loss during the last 3–6 months was calculated from the nursing homes previously registered weight of the participants. To evaluate BMI and weight loss during the last 3–6 months alone the cut-off values in MUST were used. MUAMC was calculated from measurements of mid upper arm circumference (MUAC) and TSF. MUAC and TSF were both measured according to standardized procedures described in NHANES III [16]. The values of TSF and MUAMC were compared with Symreng’s reference values [17]. TSF and MUAC were measured by the same investigator throughout the whole data collection period. The hand-grip strength was measured as previously described by Ha et al. [18], and compared with Luna-Heredias reference values [19]. The average value of three measurements of both TSF and hand-grip strength was used in analysis.

All continuous data were normally distributed as evaluated by visual inspection of a histogram and a normal Q-Q plot. Data are presented as mean ± SD. Differences in continuous variables between groups were analyzed using a two-sample *t*-test. Categorical data were tested with the chi-squared test. The statistical level of significance was set at 5%.

## Results

### Sample characteristics

**Figure 1:**
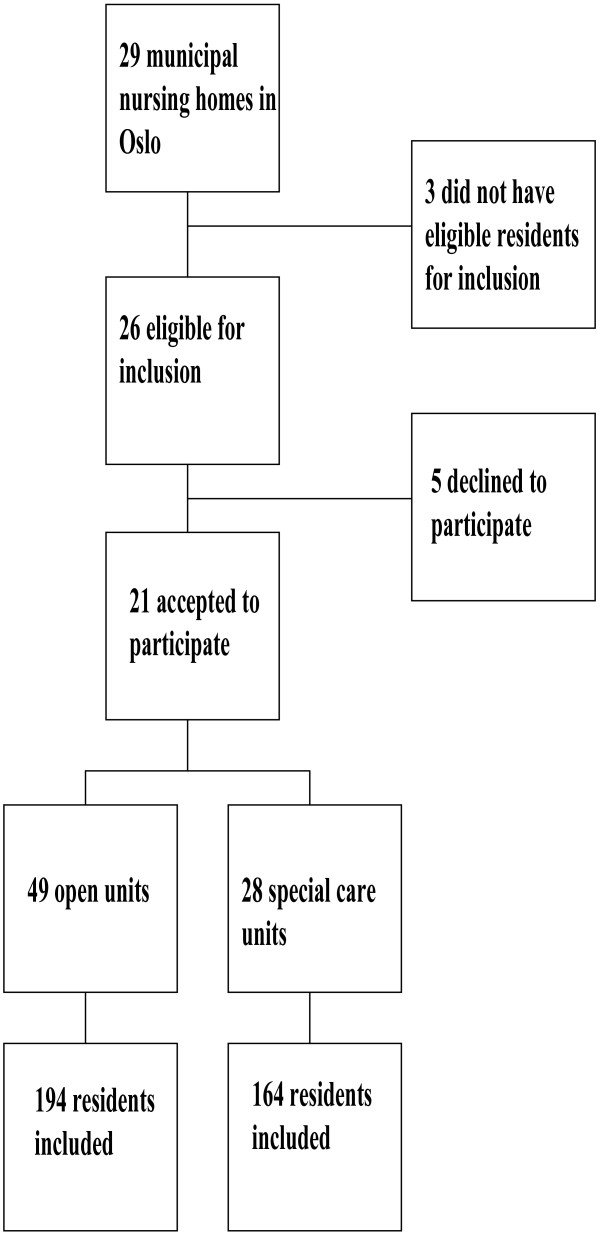
Flow chart illustrating the selection of the final sample of study participants.

Five of the 26 (19%) invited nursing homes declined to take part because of infections, other on-going projects or insufficient staff at the time of data collection. The 21 participating nursing homes had a total of 88 units. In 12/21 (57%) of the nursing homes did all units participate. The participating units were randomly chosen among the remaining 9 nursing homes. Hence we included 77/88 (88%) of the eligible units (Figure 1). In total, 404 residents were invited to participate. Of these 358 (89%) residents accepted, comprising 274 (77%) women and 84 (23%) men. The mean age was 87.1 ± 6.2 years for women and 81.6 ± 8.0 for men (p < 0.001). The residents in both the open and special care units usually came from the same catchment area prior to admission to each of the nursing homes. Regarding income each nursing home charges a fee corresponding to 75% of the resident’s pension. In the included 28 special care units, all the 164 residents had been diagnosed with dementia while the numbers with dementia and cognitive impairment in the included 49 open units were 133/194 (69%) and 61/194 (31%), respectively. There were more male residents with cognitive impairment (34%) than dementia (16%) in the open units (p = 0.006). The residents in the special care units were younger (p < 0.001) than those in the open units, 84.4 ± 6.8 and 87 ± 7.1 years, respectively.

### Nutritional assessment according to MUST

**Figure 2:**
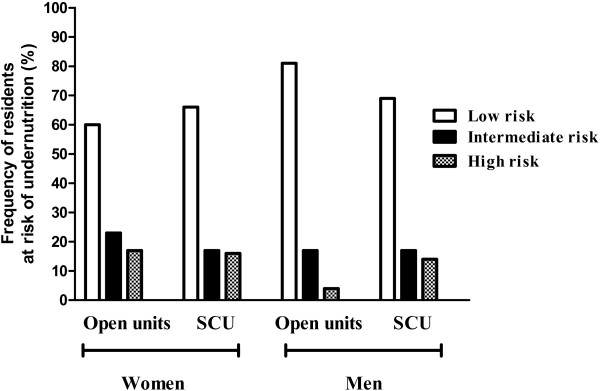
**Frequency distributions of female and male residents in various risk categories for undernutrition according to MUST and the type of residence (open units vs. special care units).** SCU – special care units.

MUST was used as a screening tool to evaluate nutritional risk, and could be scored for 309/358 (86%) of the residents. Of these 67% were classified as being at low risk, 20% were at medium risk, and 13% were at high risk of undernutrition. Figure 2 shows the nutritional status according to MUST among women and men in the two unit types. There were no significant differences in the proportions categorized as at low, middle or high risk for undernutrition between residents in open and special care units, and there were no significant difference regarding the risk of undernutrition between women and men assessed by MUST. Information about involuntary weight loss in the last 3 to 6 months could be obtained for 322/358 (90%) of the sample. Among these 80% had a weight loss < 5%, while 15% and 5% had a weight loss between 5 and 10% and > 10% respectively. There were no significant differences in the proportions of weight loss between women and men, or between residents in open and in special care units in the various categories of weight loss. Standing measurements of height and weight could be performed more frequently among residents in special care units (78%) compared to residents in open units (43%).

### Nutritional assessment according to BMI

Average BMI was similar for residents in open and in special care units. There were no statistical differences in age, height or BMI between residents with cognitive impairment and residents with dementia, and the gender proportions in the various BMI categories did not differ between open and in special care units (data not shown), hence the data for women and men are pooled. Table 1 shows the proportions of residents in various BMI categories, that is the underweight (< 18.5 kg/m^2^), normal weight (18.5–25 kg/m^2^), overweight (25–30 kg/m^2^) and those with obesity (> 30 kg/m^2^). There was no difference (p > 0.05) between the two unit types with regard to the distribution of residents within these BMI categories. The highest proportion of residents had a BMI between 20 and 25 kg/m^2^, and thus had the recommended weight according to the BMI criteria of MUST.

**Table 1:** Anthropometry and body mass index

**Parameter**	**Open units n = 194**	**Special care units n = 164**
^1^Height (m)	1.59 ± 0.09	1.60 ± 0.09
Weight (kg)	61.0 ± 13.4	61.5 ± 14.2
Body mass index (kg/m^2^)	24.0 ± 4.5	24.0 ± 4.9
^2^Body mass index (kg/m^2^) categories		
• Body mass index < 18.5	9.9	12.6
• Body mass index 18.5–25	53.0	51.6
• Body mass index 25–30	27.2	26.4
• Body mass index > 30	9.9	9.4

^1^Values are mean ± SD. ^2^Values are %. No differences (p > 0.05) between residents in open versus special care units were detected for any of the parameters.

### Nutritional assessment according to arm anthropometry

The gender proportions in the 10th and 5th TSF, MUAMC percentiles did not differ between open and in special care units (data not shown), so the data were pooled.

TSF was used as an indirect measure for subcutaneous fat depot and could be measured in 343/358 (96%) of the participants. The mean TSF in the whole sample was 13.6 ± 5.5 mm. We found that 84% had TSF within the reference range, while 8.1% and 7.8% had values between the 10th and 5th percentile and under the 5th percentile, indicating moderate and high risk of undernutrition, respectively. There were no significant differences between residents in open and in special care units, either for the mean TSF or for the residents with TSF under the 5th percentile, between the 5th and 10th percentiles or over the 10th percentile.

MUAMC was used as an indirect measure of muscle mass, and could be measured in 346/358 (97%) of the participants. As many as 92% had MUAMC within the reference range, while 5% had MUAMC between the 5th and 10th percentile, and 3% had values under the 5th percentile, which represents cut-off values for moderate and serious undernutrition, respectively [17]. There were no significant differences between residents in open and in special care units, either in mean MUAMC or in the proportion with MUAMC under the 5th percentile or between the 5th and 10th percentiles.

Hand-grip strength was used as an indirect measure of muscle strength and -function. The hand-grip strength test could be carried out by 254/358 (71%) of the residents. The residents (both women and men) in the special care units were stronger (p < 0.01) than those in the open units, with an average hand-grip strength of 15.5 ± 6.7 and 13.2 ± 7 kg, respectively. More residents in the special care units (83%) compared to the open units (72%) had a hand-grip strength of over 85% of the maximum hand-grip strength for healthy controls (p = 0.018).

## Discussion

In this study we did not detect any significant differences in the risk, assessed by the MUST screening tool or BMI, of undernutrition between residents in open units and those in special care units. Nor were there significant differences in anthropometric measures (TSF and MUAMC) between the two groups. Residents in the special care units were significantly younger and stronger than residents in open units, and standing measurements of height and weight could be performed more frequently among residents in special care units, which may indicate better mobility.

The Norwegian Directorate of Health recommends the use of the screening forms Mini Nutritional Assessment (MNA) and MUST to evaluate nutritional risk in the primary health services. A comparison of MNA and MUST showed a systematically lower prevalence of undernutrition if people were evaluated with MUST versus MNA [4]. The proportion of undernourished could therefore be underestimated in this study compared to studies that used MNA. Reference values for BMI in this study are in line with the criteria for undernutrition proposed by the Norwegian Directorate of Health for people > 70 years.

MUAMC and TSF were measured with the residents sitting due to immobility. Even if a standing position is recommended this has probably little significance [20]. Posture and arm and hand placement can change maximum hand-grip strength. It is also crucial that the individual cooperates, which can be difficult for patients with dementia or cognitive impairment.

It is unknown whether there are differences in the nutritional status of residents in open versus those in special care units. Luo and colleagues reported that residents in special care units had a significantly lower risk of having a weight loss of over 5% in the previous 30 days than residents in nursing homes without special care units [21]. Nobili and colleagues found no differences in nutritional status between the two groups on admission to a nursing home [22].

Sælbek and colleagues concluded that residents in open and special care units represented distinct nursing home populations [23]. Possibly residents in special care units may have better nutritional status because of the specially adapted offers with more carers per residents and that the carers had special knowledge of this group. However, no change in nutritional status was found in the present study between these two groups of residents, evaluated with either MUST or BMI. There were also no differences in measurements of MUAMC and TSF.

Residents in special care units display behavioural disturbances to a greater degree than residents in open units, which was found to be a risk factor for rapid weight loss in Alzheimer’s patients in a 6-year follow-up study [24,25]. Although wandering may be a predictor of weight change, a study showing wandering behaviour in 1/3 of the participants, reported that energy consumption in these was not increased, and Rolland and colleagues found that wandering was not an independent risk factor for weight loss and undernutrition [9,25]. A possible explanation is that a high level of physical activity will lead to an increased intake of food, and that food intake thereby balances the activity level. Unfortunately no information regarding food intake or the level of physical activity for the residents in our study, was available.

Gillette-Guyonett and colleagues found a significant association between weight loss and a higher score on standardized measures for the carer’s burden and stress, where behavioural disturbances contribute strongly to carers perceiving themselves as overburdened [26]. One study found that behavioural disturbances, especially irritability, restlessness and uninhibited behaviour, were strongly associated with a change in food intake [27]. Another study showed that people with major behavioural and cognitive problems had the greatest food intake at breakfast [28]. Traditional procedures at nursing homes with the least energy-rich food at breakfast and the most energy-rich food at lunch and dinner may therefore be less optimal for this group of residents.

There are some limitations to this study. The total number of participating residents was 358 and a larger sample might give better data. Moreover, according to the staff, the diagnosis of dementia should be based on the International Classification of Diseases (ICD) version 10. However, the nursing homes’ records did not always give sufficient data concerning the diagnostic procedures/tests, so misclassification of diagnosis may have occurred. We had only ethical approval to retrieve data whether they had dementia/cognitive impairment and not to re-assess their diagnoses. In line with this, other diagnoses or information about their clinical status, e.g., whether they were agitated or had other impaired functions, were not disclosed to us. Furthermore, the few residents receiving parenteral or enteral nutritional support were excluded. These factors may have an impact of their nutritional status. Of note, a criterion for living in a special care unit is a verified diagnosis of dementia. Hence, any diagnostic uncertainty would most likely affect the participants living in the open somatic units, therefore our comparison between patients living in special care units versus open units should be cautiously interpreted. In addition we have no information about the few residents who declined to participate, and can therefore not exclude the possibility that they differed in any particular way. The study was limited to long-term residents ≥ 65 years living in municipal nursing homes in Oslo, so we have no information about the nutritional status for younger residents, residents in private nursing homes or residents in nursing homes in other parts of Norway. Moreover, biomarkers of nutritional status and methods for direct measurement of body composition were not available for this study. Among the strengths of this study we would emphasize that the participation among nursing homes was high (72%) as was the attendance of the individual participants (89%). We also used robust tools for assessment of nutritional status, including validated anthropometrical methods as well as an international recommended screening tool (MUST). Collectively we therefore believe that our findings are most likely representative of municipal nursing homes in Oslo.

## Conclusions

The number of people with dementia in Norway is expected to double to approximately 135,000 by the year 2040 [29]. To our knowledge there are no studies that have estimated the prevalence of undernutrition among residents with dementia in Norwegian nursing homes. Even if this study did not find differences in nutritional status between residents in open and in special care units, we cannot exclude the fact that differences exist between these groups of residents as larger studies are required to examine the field more precisely. Such studies should include an examination of factors that have an effect on the nutritional status in the various groups of nursing home residents. This may identify which efforts for the maintenance of nutritional status that should be prioritized.

## Competing interests

The authors declare that they have no competing interests.

## Authors’ contributions

CA designed the study, collected and analysed the data and wrote the manuscript. HDA designed the study, collected and analysed the data and wrote the manuscript. POI designed the study, analysed the data and wrote the manuscript. All authors read and approved the final manuscript.

## Pre-publication history

The pre-publication history for this paper can be accessed here:

http://www.biomedcentral.com/1471-2318/13/26/prepub
